# Adenosine Neuromodulation and Traumatic Brain Injury

**DOI:** 10.2174/157015909789152137

**Published:** 2009-09

**Authors:** T.A Lusardi

**Affiliations:** R. S. Dow Neurobiology Laboratory, Portland OR, USA

**Keywords:** Adenosine deaminase, adenosine kinase, nucleotidase, nucleoside transport, caffeine, comorbidity.

## Abstract

Adenosine is a ubiquitous signaling molecule, with widespread activity across all organ systems. There is evidence that adenosine regulation is a significant factor in traumatic brain injury (TBI) onset, recovery, and outcome, and a growing body of experimental work examining the therapeutic potential of adenosine neuromodulation in the treatment of TBI. In the central nervous system (CNS), adenosine (dys)regulation has been demonstrated following TBI, and correlated to several TBI pathologies, including impaired cerebral hemodynamics, anaerobic metabolism, and inflammation. In addition to acute pathologies, adenosine function has been implicated in TBI comorbidities, such as cognitive deficits, psychiatric function, and post-traumatic epilepsy. This review presents studies in TBI as well as adenosine-related mechanisms in co-morbidities of and unfavorable outcomes resulting from TBI. While the exact role of the adenosine system following TBI remains unclear, there is increasing evidence that a thorough understanding of adenosine signaling will be critical to the development of diagnostic and therapeutic tools for the treatment of TBI.

## INTRODUCTION – ADENOSINE IN TBI

Adenosine (Ado) is a signaling molecule with widespread actions throughout the body. A Pubmed search for adenosine reviews restricted to 2008 revealed over 50 articles reviewing adenosine physiology, pathology, modulation, and therapeutic targets for subjects ranging from cardiovascular, renal, enteric, and sleep function to asthma, inflammation, and dermal wound healing. In the central nervous system (CNS), adenosine (dys)regulation is implicated in cognition, psychiatric function, Parkinson’s disease, Alzheimer’s disease, epilepsy, and hypoxia/ischemia. This review is intended to highlight the role of adenosine acutely following TBI as well as the potential adenosine-related mechanisms in co-morbidities of and unfavorable outcomes resulting from TBI, building on earlier studies [113]. 

The distribution of adenosine receptors makes Ado both an attractive target for continuing study and provides a potentially daunting complication for adenosine-based treatment strategies. There is an extensive set of tools for the study of adenosine, including agonists and antagonists of varying selectivity and specificity, transgenic mice, and functional assays. Several adenosine-related therapeutic candidates are either currently available or in clinical trials [11, 52], potentially easing the regulatory burden for subsequent applications. However, the ubiquitous nature and diverse effects of adenosine signaling requires particular attention to drug delivery and activation, and the potentially significant side effect profile.

Adenosine is a potent neuromodulator, acting at CNS synapses to restrict synaptic activity *via* four known g-protein coupled receptor subtypes, reviewed in [11]. Adenosine receptors are found throughout the brain [15], and are implicated in diverse neurological functions and pathologies [48]. In addition to its role as a signaling molecule, the nucleoside Ado is an intermediary in a metabolic pathway that includes the nucleobase adenine, the nucleotide ATP (a primary energy substrate), and the second messenger cyclic adenosine monophosphate (cAMP) (Fig. (**1**)), which further highlights the varied consquences of Ado dysregulation. The neuroprotective role of Ado is well established in inflammation, ischemia/reperfusion injury, and asthma [90] as well as in diverse CNS diseases [20]. Unlike excitatory and inhibitory amino acids with an “all or none” effect, Ado acts in the CNS as a modulator [119], which may be a key factor reducing negative side effects such as those found with NMDA receptor antagonists [57].

Traumatic brain injury is a significant health burden in the United States; the US Centers for Disease Control estimated 1.4 million TBIs per year in 2001 [81]. In a recent survey of Iraq war veterans, 15% of returning soldiers reported a mild TBI; of those, 48% had symptoms of post-traumatic stress disorder [65]. Additional lasting effects significantly associated with a brain injury are chronic pain [102], fatigue and other sleep disturbances [141], cognitive problems [78], anxiety [98], and epilepsy [23, 122]. While these symptoms subside for many patients, they can persist for a lifetime of disability [66, 105]. 

## NEUROPHYSIOLOGY OF THE ADENOSINE SYSTEM

The A_1_ and A_2A_ receptors are widely expressed in brain, with high adenosine affinity (~100nM [38]), and complementary actions. The A_1_ receptor is a Gi/Go coupled metabotropic receptor, acting to inhibit adenylyl cyclase and cAMP production, with uniform expression throughout the CNS [16, 37]. It is generally inhibitory at synapses, activating K^+^ and Cl^-^ channels and inhibiting P- and N-type voltage gated calcium channels. The A_2A_ receptor is a Gs coupled metabotropic receptor, activating adenylyl cyclase and cAMP production (Fig. (**2**)). While RT-PCR studies show expression throughout the brain [37], it is preferentially expressed in the striatum, nucleus accumbens, and thalamus [118]. A_2A_ receptors interact with A_1_ receptors, forming functional heteromers [31], as well as with several excitatory receptors, notably the dopamine [8, 44] and glutamate systems [121, 135]. Free adenosine in the brain (the “tone”) is typically in the nanomolar range [11, 83]. Adenosine is increased locally to millimolar levels during low frequency synaptic activity [43], acting primarily *via* the A_1_ receptor as a presynaptic inhibitor of excitatory amino acid release and postsynaptically to maintain hyperpolarization [34]. Adenosine appears to act as the unifying signaling molecule in studies of the molecular basis of learning [34]. It acts as an autocrine signaling molecule at the tetanized synapse, enhancing synapse strength *via* A_2A_ receptor activation [4]. It acts as a paracrin signal *via* a calcium wave in the astrocytic syncitium, acting distant from the tetanized synapse to achieve heterosynaptic depression by A_1_ receptor activation [58]. In addition to their role at the synapse, astrocytes release Ado at endothelial cells, causing vasodilation *via* A_2A_ receptor activation, which enhances local circulation and provides the additional metabolic support rquired during intense synaptic stimulation [61].

The low-affinity (micromolar [38]) A_2B_ and A_3_ receptors are also widely expressed in brain, though at low levels [37]. Their low affinity for adenosine makes them likely mediators of excessive adenosine signaling, such as occurs in trauma, but there is little research on their specific roles. Like the A_1_ and A_2A_ receptors, the A_2B_ and A_3_ receptors have complementary actions; the A_2B_ receptor is Gs coupled, and the A_3_ Gi/Gq coupled (Fig. (**2**)). Unlike the A_1_ and A_2A_ receptors, their expression seems to be mainly astrocytic. Stimulation of the A_2B_ receptor rapidly triggers interleukin-6 production, making this a likely step in the inflammatory response following trauma [140]. A_2B_ receptors are upregulated following ischemic preconditioning, again suggesting a primary role in endogenous neuroprotective mechanisms [149]. The role of the A_3_ receptor is more controversial [10]. Studies have shown that A_3_ receptor activation is protective in astrocytes [17]. In neurons, a more complicated response has been revealed, with A_3_ receptor activation protecting CA1 neurons during short duration oxygen-glucose deprivation, yet causing damage during long-duration depriviation [116]. 

## PATHOLOGY OF THE ADENOSINE SYSTEM AFTER TBI

The study of TBI is unusual in that the invasive procedures required to stabilize and monitor a severely injured patient have facilitated clinical studies that would be impossible in other injuries or chronic diseases. Intracranial pressure (ICP) has long been recognized as an indicator of TBI severity [56, 97]. ICP monitoring is an important component of a multi-modal monitoring procedure in the neurological intensive care unit [145]. As it requires insertion of a catheter into the ventricle, ICP monitoring allows regular monitoring of CSF. Consequently, we have an extensive understanding of changes to adenosine and its metabolites following human TBI.

### Cyclic AMP

3'-5'-cyclic adenosine monophosphate (cAMP) is a key component of the adenosine metabolic pathway (Fig. (**2**)). cAMP is a second messenger regulated by G-protein coupled receptors which serve to rapidly couple extracellular signals to intracellular responses. cAMP is is modulated by diverse extracellular signals, including hormones, dopamine, glutmate, and adenosine. Current understanding of cAMP demonstrates the functional diversity of cAMP signaling [5] and highlights its role in synaptic plasticity [2, 39]. Specificity in cAMP signaling may be due to cellular compartmentalization [110, 148], though this is not yet well understood in brain cells. 

Early studies of the second messenger cAMP were based on the hypothesis that cAMP is a metabolic regulator. Clinical studies show that depth of coma is correlated to reduced cAMP in CSF, falling as low as 1.5 nM during grade V coma, with a steady improvement correlated to coma depth, to nearly normal levels (20 nM) upon reaching grade I [45, 124]. Of note, in these studies plasma levels of cAMP remained normal (9-19 nM), even when CSF levels were < 6 nM, suggesting that the CSF cAMP measures reflect changes specific to the brain, and not a systemic reduction [45]. Regulation of CSF cAMP levels has been demonstrated in many neurological diseases. Eight-to-twelve hours following cerebral infarction, increased cAMP has been measured [100], but levels are depressed by 3 days [24]. Following epileptic seizure, depressed cAMP levels were measured for 3 days [100], and are chronically low in patients with multiple sclerosis [94]. These measurements of cAMP provide a valuable diagnostic tool, as well as insight into the evolution of brain injury. 

### Cerebral Blood Flow

Oxygen and glucose are critical to brain function; continuous cerebral blood flow (CBF) is necessary as there are no energy stores in the brain. The mechanisms of CBF and metabolism have been described in detail [142]. Autoregulatory mechanisms in the brain circulation can compensate for minor or short term disruptions in blood flow, pressure, and/or volume. Brain swelling is a common consequence of TBI; as the volume within the intact skull is fixed, such swelling results results in compression of the ventricles (reduction in CSF volume) and vasoconstriction (reduction in blood flow). While clinical monitoring of patients with severe head injury has revealed wide fluxuations in CBF, oxygen demand remains consistently low [108], even during hyperperfusion [33]. Kochanek *et al.* measured CSF levels of adenosine and cerebral oxygen use to test the hypothesis that adenosine could account for this uncoupling, as adenosine causes vasodilation *via* A_2A_ receptors on endothelial cells, and represses synaptic activity through the A_1_ receptor on neurons. They found that increased adenosine was associated with depressed arterio-jugular venous oxygen difference and increased risk of death [32, 73]. Using microdialysis, Bell *et al.* demonstrated increased adenosine and cAMP levels in the cortex after TBI during secondary oxygen desaturation, correlating these increases to increased glutamate and lactate [14], further supporting a role for adenosine release as a critical mediator of the cerebral response to TBI.

### Cellular Metabolism

Closely related to cerebral blood flow is cellular metabolism, the means by which brain cells produce their primary energy substrate ATP. The first step is glycolysis, in which glucose is converted to pyruvate:

Glucose + 2NAD^+^ + 2ADP + 2P_i_ >> Pyruvate + 2NADH + 2H^+^ + 2ATP + 2H_2_O.

Under physiologic conditions, pyruvate and NADH become the substrates for aerobic metabolism by the citric acid cycle, oxidizing NADH to NAD+ as well as generating ATP. Under anaerobic conditions, NAD+ is replenished by the enzyme lactate dehydrogenase:

$$\text{Pyruvate}+{\text{NADH}}^{+}+{\text{H}}^{+}\gg \text{Lactate}+{\text{NAD}}^{+}.$$

Lactic acid is readily measured in the CSF, and a clear indicator of hypoxic conditions in the brain.

Hypoxia and ischemia are common co-morbidities of TBI [29, 55, 67], and their treatment is the focus of significant clinical literature [28, 51, 63, 64, 95, 150]. Not all cerebral metabolic crisis is due to ischemia however; combined PET scans and microdialysis showed significant increases in the lactate/pyruvate ratio in the absence of ischemia [143], suggesting increased anaerobic metabolism. 

Breakdown of ATP in metabolically stressed tissue is a potentially significant source of adenosine; clinical measurements following TBI demonstrate lasting alterations in the adenosine metabolic pathway. Cerebralspinal fluid (CSF) samples taken following severe TBI show significant elevations in adenosine in both adults [32] and children [120, 129]. Further studies have shown that components of the adenosine metabolic pathway including cAMP, adenosine, inosine, hypoxanthine, and xanthine are all transiently upregulated during secondary hypoxic periods in both clinical [14], and experimental [13] settings. Clinically, Ado levels are typically highest at early time points, though late elevations (> 72 hours following TBI) have been noted in children [120], and likely reflect the progression of secondary injury. Using microdialysis, Bell *et al.* demonstrated increased adenosine and cAMP levels in the cortex after TBI during secondary oxygen desaturation, correlating these increases to increased glutamate and lactate [14]. In rodent models of fluid percussion injury, transient decreases in ATP (with recovery by 24 hours) followed similar kinetics of adenosine increase; however, when FPI was combined with secondary ischemia, ATP remained depressed at 24 hours [6]. While these studies make it clear that the adenosine system is altered by TBI, it is not yet clear whether this increased adenosine reflects endogenous neuroprotection mechanisms or is a byproduct of ATP breakdown.

## MODULATION OF ADENOSINE RECEPTOR ACTIVITY

Direct modulation of adenosine (up to 30-fold) in response to stimuli has been measured in multiple model systems [83]. Neuroprotection by adenosine receptor modulation has been demonstrated in several model systems, including hypoxia/ischemia [123, 133], epilepsy [18, 101], Parkinson’s disease [27, 69, 130], inflammation [26, 60] among others [27, 38, 119]. They play a key role in many organ systems, however, and have been the target of several drug treatments for respiratory disease [137, 139, 147], cardiovascular disease [40, 111], and hepatic injury [12]. While no clinically effective neuroprotective strategies based on adenosine receptor modulation have yet emerged, modulation of adenosine receptor activity has provided insight into the diverse roles of adenosine in neural function and dysfunction. 

### Agonist/Antagonist Studies

*In vivo* studies of adenosine receptor modulation in TBI have demonstrated modest therapeutic improvements. The non-selective Ado agonist 2-chloro-adenosine (2CA), active at A_2B_ > A_1_ > A_2A_ receptors (IUPHAR database [59]), has been used in several experimental models. Kochanek *et al.* have demonstrated that intraparenchymal injection of 2CA increases CBF in the rat brain in a broad, dose-dependent and persistent manner [75]. Administration of 2CA by intrahippocampal injection after CCI restored CBF, more effectively in moderate injury than in severe injury [74]. By other measures, however, the effects of 2CA treatment are mixed. Pre-treatment with 2CA by intra-cerebral ventricular (ICV) injection demonstrated partial restoration of [Mg^2+^] levels, and a small, but non-significant, improvement in neuroscore at 1 and 7 days following injury [62]. Post-CCI, intrahippocampal injection of 2CA resulted in improved wire-grip scores but no improvement in memory tasks or hippocampal cell survival [138]. Post injury treatment with the A_1_ receptor agonist CCPA improved the CA3 cell count, but had no significant effects on learning and behavior measures [138], while the A_1_ receptor antagonist DPCPX and its solvent DMSO both resulted in increased lesion volume and reduced balance beam activity [138]. Together, these results reinforce the role of adenosine receptors as neuromodulators, with effects specific to the selected outcome measure. Additional studies with longer survival times are also likely to demonstrate more definitive effects of adenosine receptor antagonism and antagonism. 

Interpretation of drug studies is complicated by our evolving understanding of receptor pharmacology as well as different methods of assessing agonist/antagonist kinetics. Within these limits, relative adenosine affinity must also be considered; A_1_ and A_2A_ receptors have a much higher Ado affinity (10 – 100 times greater [38]) than A_2B_ and A_3_ receptors, so modulation of the A_2B_ and A_3_ receptors may only influence the most extreme adenosine response, and may define the difference between beneficial and harmful adenosine activity. The presence of A_1_/A_2A_ heteromers further complicates the interpretation of activation/inhibition studies. The use of adenosine receptor knockout mice allows the examination of the specific receptor effects without the confounding effects of invasive drug delivery methods and non-specific vehicle effects (eg, DMSO). Adenosine receptor knockouts have a relatively normal phenotype, making them an attractive tool for the study of adenosine regulation following TBI [49]. Knockout of the A_1_ receptor leads to lethal status epilepticus following cortical contusion injury [76], reinforcing the neuroprotective role of the A_1_ receptor as a synaptic inhibitor. Concurrent measurement of cAMP in this model would be interesting, as A1 receptor activation inhibits adenylyl cyclase and A1 knockout would be expected to enhance cAMP production (Fig. (**2**)), giving some additional insight into the causality of the cAMP-adenosine cycle. Knockout of the A_2A_ receptor is neuroprotective in the CCI model [87], with improved neuromuscular behavior, reduced tunel staining, and reduced glutmate release. Perhaps most interestingly, the pro-inflammatory cytokines TNF-α and IL-1β were significantly decreased by 24 hours following injury in the A_2A_ knockout mice when compared to wild-type mice. Though the specificity of these effects is still unclear, these results further illustrate the complexity of adenosine signaling and the interdepencence of adenosine signaling, neural tissue, the immune response, and the cerebrovascular system; any therapy affecting one parameter is likely to have an affect on the others.

### Caffeine

Caffeine is a well-known stimulant with a primary inhibitory activity at the A_1_ and A_2A_ receptors. Coffee, tea, and soda are common sources of dietary caffeine, containing 40-120 mg/serving, and in excess of 200 mg in “energy” drinks. Elevated caffeine in CSF has been correlated to improved outcome in clinical studies; at 6 months following TBI, elevated caffeine was a stronger predictor of outcome than Glasgow Coma Score or alcohol on admission [126]. In modeled TBI, acute pre-treatment with caffeine (50 – 150 mg/kg) worsens outcome by all measures studied, including mortality, behavior, edema and blood-brain barrier breakdown, and peroxidase activity [3, 86]. In contrast, chronic pre-treatment with caffeine resulted in improvements in neuroscore, edema, apoptosis, CSF glutamate, and markers of the inflammatory response (CD45, TNF-α, and IL-1) [86]. Of note, chronic caffeine treatment resulted in increased A_1_ receptor mRNA expression [86]; a corresponding increase in A_1_ receptor expression is likely a significant contributor to the protective effects of chronic caffeine. 

The effects of caffeine are not limited to the adenosine receptors. Caffeine stimulates IP-3 receptor mediated intracellular calcium release; calcium activates adenylyl cyclase, which catalyzes ATP into cAMP. Mechanical deformation of neural cells causes a significant acute rise in cytosolic [Ca2+] [82, 91, 125]; however, following stretch, it is not possible to to elicit this calcium response [146], suggesting that acute pre-treatment with caffeine may cause rapid and sustained increase in neuronal calcium, disrupting ionic homeostasis, and sensitizing the brain to a subsequent injury. The combined actions of caffeine to enhance cAMP production *via* adenylyl cyclase activation may have a role in the effects of acute caffeine treatment; however, evidence in the literature is contradictory. Clinically, cAMP increases during oxygen desaturation after TBI [14], but does not change during secondary swelling [73]. In a rodent model of CCI, no acute changes were measured in cAMP [13]. In contrast, following lateral fluid percussion injury, not only were significant reductions in cAMP measured, but phosphodiesterase inhibition with rolipram restored cAMP levels, reduced negative histopathological findings, and decreased the inflammatory response [7]. While the precise role of caffeine in the severity and recovery from TBI remains unknown, its wide use and clear effects in modeled TBI make it a potential confounding factor in clinical evaluation and treatment.

## ADENOSINE REGULATION

Adenosine levels in the brain are typically in the range of 30-300 nM. Key Ado modulators include 5’-nucleotidases, adenosine deaminase (ADA), and adenosine kinase (ADK) (Fig. (**2**)). While neurons and glia both contribute to the maintainence of adenosinergic tone, expressing many similar enzymes, there is evidence that they respond to stimuli by different pathways [110]. Further, regionally distinct expression and activity of each of these enzymes suggests that the adenosine tone may differ by region [112]. Adenosine involvement in the sleep/wake cycle is well established (recent reviews include [80, 96, 132]), and activity of these adenosine modulators in the sleep regions of the brain follow similar diurnal patterns [92]. Further studies have demonstrated the effects of age on adenosine modulator activity, and shown that, while ADA does not vary with age, 5’nucleotidases and ADK both increase significantly with age [93]. While there is little direct study of these adenosine modulators in the context of TBI, their rapid and direct influence on the levels of adenosine in the brain make them a likely influence on outcome after TBI.

### 5’-Nucleotidases

Located on the cell surface (ecto-) and in the cytosol (cytosolic, endo-), 5’-nucleotidases act to hydrolyze ATP, AMP, and ADP to Ado [22]. Two commonly studied nucleotidases are CD39 (also known as apyrase and nucleotide triphosphate diphosphohydrolase) and CD73. CD39 hydrolyzes ATP to ADP and AMP, while CD73 hydrolyzes AMP to Ado. In particular, increases in CD73 function, mRNA, and protein have been shown in endothelial monolayers, as early as 8 hours following the onset of hypoxia or reperfusion [88, 90], suggesting that it may be a component of the endogenous response leading to ischemic preconditioning [47]. Models of pilocarpine-induced epilepsy suggest that enhanced ecto-nucleotidase activity may have a significant role in the “silent” phase of epileptogenesis [144]. *In vivo* studies examining the cellular response to a cortical stab injury (CSI) show a biphasic regulation of CD73: by 4 hours following the CSI, AMP hydrolysis is significantly reduced in the region of the stab wound [104], but by 15 days it has increased significantly in all regions of the brain considered [103]. In contrast, CD39 activity changed little, and only in the region of the CSI [104].

### Adenosine Deaminase

Adenosine Deaminase (ADA) catalyzes the conversion of Ado to inosine (reviewed in [46]), and can be located either in the cytosol or on the extracellular surface of the plasma membrane of neurons, glia, and endothelial cells [30, 99, 117]. ADA acts rapidly during ischemic events to decrease local Ado [109]. As Ado has anti-inflammatory properties [26, 60], it has been hypothesized that inhibition of ADA might increase abient Ado, reducing inflammation. The ADA inhibitor FR234938 reduced plasma levels of the pro-inflammatory cytokine TNF-α and elevated the anti-inflammatory cytokine IL-10 in response to lipopolysaccharide treatment [77]. Further, treatment with the ADA inhibitor deoxycoformycin was protective following permanent focal ischemia, with a notable reduction in swelling [89]. Of interest, colocalization of ADA, the A_1_ receptor, and the cytokine CD26 (also known as dipeptidyl peptidase IV, DPP-IV) has been noted in many organs and across species [1, 16]. CD26 interacts directly with ADA on T-cells [70] as an active component of the immune response, and the colocalization of this trio of proteins on endothelial cells [79] may have a role in the regulation of the blood-brain barrier following trauma. While the mechanisms of these interactions are not clear, this colocalization and conservation across species suggest a causal mechanism.

### Adenosine Kinase

In the developing brain, adenosine kinase (ADK) expression is primarily in the cytosol of neurons [134]. As the brain develops, however, ADK expression shifts to the cytosol of astrocytes; extracellular Ado is taken up by astrocytes *via* passive and facilitated diffusion, where it is phosphorylated into AMP by ADK. While it has not been studied directly in TBI, ADK regulation of adenosine has been implicated in many neurological disorders [20]. In acute injury such as ischemia, ADK upregulation is associated with poor outcome [115], while reduced ADK improves outcome [114], reviewed in [19]. Chronic diseases (such as epilepsy) have been associated with astrogliosis and ADK upregulation across multiple models, including kainic acid induced [42], kindled [85], and astrogliotic [84], reviewed in [21]. The additive effects of ADA and ADK inhibition [128] may provide an additional avenue for therapies targeting adenosine modulators.

### Adenosine Transport

The third major system for adenosine clearance is nucleoside transporters, reviewed in [72]. In microdialysis studies, larger increases in ambient adenosine were measured *in vivo* in response to treatment with uptake inhibitors than were measured with ADA inhibitors, without the concurrent rise in adenosine metabolites inosine and hypoxanthine [9]. In a model of cerebral contusion, treatment with the transport inhibitor propentofylline increased expression of the neuroprotective peptide basic fibroblast growth factor [25]. As ethanol is a common factor in TBI, a potential additive role of adenosine in the cardiovascular response was measured in a swine model of TBI; however, no significant protection was measured with adenosine transport inhibition [41]. Adenosine uptake inhibition remains an untapped source of potential neurprotective strategies [106].

## ADENOSINE REGULATION AND TBI CO-MORBIDITIES

While many TBI survivors regain gross motor and mental functions, comorbidities often remain throughout life. Studies following mild and moderate TBI survivors show improvements in community integration, cognitive and neuropsychiatric measures over 6-12 months [107], 5 years [136], and 22 years [105]. However, impairments persist, and there is significant opportunity for therapeutic intervention. Chronic pain is a common result of TBI, even after mild injury [102]. Adenosine modulation has been widely implicated in inflammation [26, 53, 60] and analgesia [38, 72, 127]. Anxiety is one of many neuropsychiatric comorbidities of TBI [98], with clear correlates to adenosine dysregulation [35, 54, 68]. Post traumatic epilepsy has a long latency, often decades, and is particularly common following severe injury in both pediatric [131] and adult [50] populations. Even in individuals without clinical seizure manifestations, epileptiform activity has been reported [122]. In an A_1_ receptor knockout mouse, TBI led to lethal status epilepticus [76], suggesting a clear role for the A_1_ receptor in seizure suppression. To date, two models of spontaneous seizure development following lateral fluid percussion injury (LFPI) have been published, with different characteristics. D’Ambrosio *et al.* reported rapid onset of high frequency, short duration cortical seizure activity, accompanied by generally mild behavior manifestations within the first 8 weeks of (1-2 on a the 1-5 Racine scale)[36]. Kharatishvili *et al.* report a latency of 2-12 months before seizure onset of seizures, similar to the latency observed clinically following TBI, with seizure activity lasting nearly two minutes [71]. Further studies implicate adenosine dysregulation in epileptogenesis and epilepsy, reviewed in [21]. Taken together, these results suggest that adenosine modulation a source of therapeutic potential across the spectrum of traumatic brain injury and recovery. 

## CONCLUSION

There is strong evidence that adenosine (dys)regulation plays a role in the brain following TBI. It is still not clear whether Ado is an endognous neuroprotective measure or a byproduct of stressed cellular metabolism; the truth is likely in between, and linked to injury severity. Current studies highlight the potential of adenosine to act in both protective and detrimental pathways; the multi-factoral pathologies of TBI and effects of adenosine signaling may mean that adenosine is not a practical target for neuroprotective therapies following TBI. Whether the adenosine system emerges as a neuroprotective target, it is clear that adenosine has a role in the evolution of neural recovery after traumatic brain injury, and a thorough understanding of its influences in the CNS will be critical in the development of diagnostic and therapeutic tools.

## Figures and Tables

**Fig. (1):**
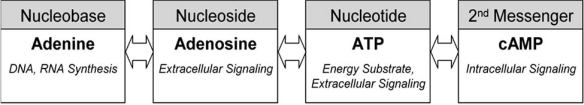
Adenosine and its metabolites are active at all levels of cellular function.

**Fig. (2):**
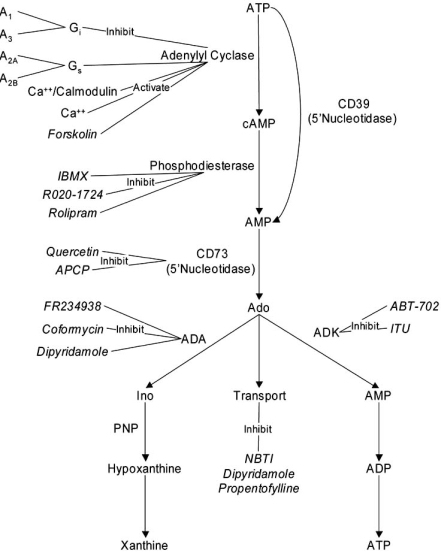
Adenosine and metabolites regulated in response to TBI. Compounds in italics are exogenous drugs discussed in the text.

## ABBREVIATIONS

2CA: = 2-Chloroadenosine
Ado: = Adenosine
ADA: = Adenosine Deaminase
ADK: = Adenosine Kinase
ADP: = Adenosine Diphosphate
AMP: = Adenosine Monophosphate
ATP: = Adenosine Triphosphate
cAMP: = Cyclic Adenosine Monophosphate
CCI: = Cortical Contusion Injury
CCPA: = 2-Chloro-N(6)-Cyclopentyladenosine
CNS: = Central Nervous System
CSF: = Cerebral Spinal Fluid
CSI: = Cortical Stab Injury
DMSO: = Dimethyl Sulfoxide
DPP-IV: = Dipeptidyl Peptidase IV
ICV: = Intra-Cerebroventricular
LFPI: = Lateral Fluid Percussion Injury
NMDA: = N-methyl-D-aspartic acid
TBI: = Traumatic Brain Injury
